# Blockchain Based Secure Routing and Trust Management in Wireless Sensor Networks †

**DOI:** 10.3390/s22020411

**Published:** 2022-01-06

**Authors:** Saba Awan, Nadeem Javaid, Sameeh Ullah, Asad Ullah Khan, Ali Mustafa Qamar, Jin-Ghoo Choi

**Affiliations:** 1 Department of Computer Science, COMSATS University Islamabad, Islamabad 44000, Pakistan; fa19-rse-041@student.comsats.edu.pk (S.A.); sp19-rcs-012@student.comsats.edu.com or; 2 School of Computer Science, University of Technology Sydney, Ultimo, NSW 2007, Australia; 3 School of Information Technology, Illinois State University USA, Normal, IL 61761, USA; sullah@ilstu.edu; 4 Department of Computer Science, College of Computer, Qassim University, Buraydah 52571, Saudi Arabia; al.khan@qu.edu.sa; 5 Department of Information and Communication Engineering, Yeungnam University, Gyeongsan 38541, Korea

**Keywords:** authentication, blockchain, Rivest–Shamir–Adleman, secure routing, smart contract, trust evaluation, wireless sensor network

## Abstract

In this paper, an encryption and trust evaluation model is proposed on the basis of a blockchain in which the identities of the Aggregator Nodes (ANs) and Sensor Nodes (SNs) are stored. The authentication of ANs and SNs is performed in public and private blockchains, respectively. However, inauthentic nodes utilize the network’s resources and perform malicious activities. Moreover, the SNs have limited energy, transmission range and computational capabilities, and are attacked by malicious nodes. Afterwards, the malicious nodes transmit wrong information of the route and increase the number of retransmissions due to which the SNs’ energy is rapidly consumed. The lifespan of the wireless sensor network is reduced due to the rapid energy dissipation of the SNs. Furthermore, the throughput increases and packet loss increase with the presence of malicious nodes in the network. The trust values of SNs are computed to eradicate the malicious nodes from the network. Secure routing in the network is performed considering residual energy and trust values of the SNs. Moreover, the Rivest–Shamir–Adleman (RSA), a cryptosystem that provides asymmetric keys, is used for securing data transmission. The simulation results show the effectiveness of the proposed model in terms of high packet delivery ratio.

## 1. Introduction

A Wireless Sensor Network (WSN) plays an important part in the growth of various applications such as healthcare, the military, industrial surveillance, etc., [1,2,3]. In this self-organized network, Sensor Nodes (SNs) with limited energy, storage and computational capabilities are randomly distributed [4,5,6]. The SNs monitor different factors, which are wind, humidity, temperature, etc., and then forward the data to the Base Stations (BSs) [7].

One of the major issue in WSNs is security threats. The reason is that SNs are resource constrained and can be easily compromised [8,9]. Generally, there are two types of attacks that are performed in the WSNs. In external attacks, the attackers take control over the SNs to perform malicious activities, whereas, in internal attacks, SNs behave selfishly to preserve their energy and storage. Consequently, identifying and removing the malicious nodes from the network are crucial aspects [10].

The blockchain technology is an option to resolve the aforementioned issues by introducing smart contracts where all agreements in the system are written. It was introduced in 2008, and it consists of nodes that keep track of the state of the distributed ledger. In general, there are three types of blockchain network, which are public, private and consortium [11,12]. The public blockchain is fully decentralized where any node can enter and become part of a fully decentralized network. The private blockchain is a permission based network where only the selected nodes can participate. The consortium blockchain is a semi-decentralized network, which is managed by multiple organizations. The miners in the blockchain verify the transactions through consensus [13,14]. Different consensus algorithms are used in the network, which are Proof of Authority (PoA), Proof of Work (PoW), Proof of Stake (PoS), etc. In PoW, the nodes solve a mathematical puzzle for the selection of miner nodes in the network, The node that finds the puzzle solution first will add a new block in the blockchain. This puzzle solving requires high computational cost. In PoA, the blocks and transactions are validated by preselected nodes, called validators. Therefore, high computational capabilities for the selection of miners are not required. In PoS, the miners with the most coins validate and mine the blocks.

A blockchain is an effective way to keep a record of transactions between several groups in a distributed manner [15,16]. As the blockchain is immutable, no one can tamper with the data. In a blockchain, the transaction data are secure, as the blocks are linked by the hashes [17]. The hashes of the Merkle tree and previous blocks reside in the block header, whereas the transactions are present in the block body [18].

Without authentication, the intruders utilize the network resources to forge the benign nodes’ identities and locations [19,20]. The presence of intruders in the network has a negative impact on the routing mechanism. The intruders alter the data and transmit incorrect information of the route, which degrade the network’s performance [21,22]. To solve the aforementioned problems, we propose a secure routing mechanism using blockchain based encryption and trust evaluation. The contributions of our paper are given below.

1. The malicious SNs in the network are identified considering three factors: Forwarding Rate (FR), Response Time (RT) and Delayed Transmission (DT).
2. A routing mechanism is proposed that ensures real time and energy efficient data delivery from SNs to BSs. The ANs act as relay nodes in the data delivery.
3. Secure and reliable data delivery is ensured using the RSA technique.

The rest of the paper is structured as follows. Section 2 presents the related work. The problem statement and system model are presented in Section 3 and Section 4, respectively. The simulation results are discussed in Section 5. The paper is concluded in Section 8.

## 2. Related Work

In this section, a literature review of different papers is discussed on the basis of their contribution. Table 1 presents the summarized literature review of different papers.

### 2.1. Trust Evaluation of Sensor Nodes

In [10], the authors propose a trust model utilizing a blockchain to ensure secure localization. The locations of the unknown nodes are determined using the trust values of the benign nodes. The trust values are the aggregation of the behavioral and data based trust. In [19], the authors propose a trust model to prevent the involvement of malicious nodes to ensure traceability and transparency. The credibility of the nodes is computed on the basis of successful and unsuccessful communications. The authors in [20] propose a secure range free localization algorithm where the node’s location is computed based on the degree of connectivity between SNs. The trust values of the benign nodes are determined by considering mobility, remaining battery, reputation value [23] and a neighbor node list.

### 2.2. Nodes’ Authentication

In [24], the authors present an IoT authentication protocol using a blockchain. In this protocol, a sink node is placed in the network’s center. At every level, the sink node broadcasts a hello message and nodes respond to the sink node with their identities. Furthermore, the authors in [25] propose a secure key management mechanism using a blockchain, which performs two operations between nodes: registration and cluster formation. In the proposed mechanism, the BS acts as a centralized party that assigns a unique identity to each node, and generates a pair of public and private keys. In [18], the authors propose an authentication and trust model to attain confidentiality using cryptography, digital signature and peers’ identity information. A public key infrastructure is used to perform the authentication of nodes. In authentication, the node submits payload credentials, which comprise a master public key and a secondary key. As privacy and security of the network depend on the information that exists in the blockchain, therefore, only the registered nodes can add new blocks in the network. Moreover, the authors in [26] propose an IoT framework where smart sensors control the activity of all nodes. The tractability of each node requires nodes’ registration in the blockchain. In the framework, some nodes act as the miner nodes to validate the transactions. The system proposed in [27] ensures the authenticity of data using the blockchain. Moreover, it provides the mechanism to securely store the data of the overall network.

### 2.3. Secure Routing in Networks

The authors in [28] present a framework called intrusion prevention for mobile IoT devices to provide reliable data routing based on a blockchain. The proposed framework is categorized into two phases. Firstly, every node stores its neighbors’ information in the routing table. Using the uncertainty principle, the cluster heads are selected. Secondly, the authors present a security model that improves the network reliability based on the blockchain. Furthermore, the authors in [29] propose a blockchain based encryption and localized routing scheme to discover a route. Moreover, blockchain technology is used for data security that separates the data into blocks. Furthermore, a blockchain based contractual routing protocol is introduced that establishes trust between IoT vendors and cooperators during data transmission. The proposed system comprises a multi-hop network where the sets of source, intermediary, destination and gateway devices are present. In the blockchain contractual routing, each source node uses a smart contract to request a route from gateways or destination nodes within a specific time [30]. Moreover, the authors in [31] propose a routing scheme using blockchain and reinforcement learning to enhance both the routing efficiency and the security of WSNs. The proposed scheme consists of two parts that are the blockchain and routing network. Moreover, there are three types of nodes: server, terminal and routing. The routing nodes are connected with a terminal node that receives the packet from other nodes. The source terminal sends the packet to the target terminal with the help of intermediary nodes, called routing nodes. Furthermore, the server nodes aggregate the data packets. Blockchain technology ensures fairness and tractability of the transactions. A consensus mechanism, PoA, is selected that efficiently processes the transactions performed in the network.

### 2.4. Lightweight Blockchain

The proposed framework in [32] consists of four layers: light chain, cache, storage and Application Programming Interface (API). The light chain layer combines several modules of the blockchain. The cache layer contains useful pending blocks and local operations that are managed by a light chain. The storage layer provides storage to the upper layer. The API layer provides services to the industrial applications and extracts functionalities of the light chain and cache layer.

The authors in [33] present a hierarchical structure that consists of the IoT, fog and cloud layers. The blockchain is deployed on the cloud layer while the fog layer contains a smart gateway. In the IoT layer, different underwater IoT devices transfer data packets to the cluster heads and then cluster heads forward them to gateways.

In the proposed system of [34], aggregated information is used to overcome the communication cost by utilizing blockchain technology. The authors in [35] present an optimized policy using Tangle [36] and blockchain technologies for sampling rates. The goal is to lessen the age of information in the IoT network for reliable data exchange. Moreover, this model provides the updated information to the users after validation. In the blockchain applications, mobile devices face issues generated by the PoW puzzle in the mining procedure. The reason is that PoW requires high storage and computational capabilities to solve the puzzle [37].

### 2.5. Data Storage

The authors in [38] propose a mechanism in which the SNs are incentivized and motivated to store the data of the network. The data are organized in the form of a block where each block is chained with other blocks to form a complete blockchain. Network nodes that store the data on the blockchain are rewarded in the form of digital currency. If provable data possession is valid, then a new block of data is added in the blockchain and a reward for data storage is obtained. The authors in [39] present a rolling blockchain that uses smart cars as the nodes of the WSN. The reliability of the network is analyzed by the number of nodes and their connections. The blocks are added in the blockchain after being verified from the nodes. Each node has a neighbor node list that uses minimum power in transceiving information to neighbors.

**Table 1:** Literature review.

Problems Already Addressed	Solutions Already Proposed	Validations Already Done	Problems to Be Addressed	C1	C2	C3
Incorrect location estimation and energy dissipation	Node’s trust values are based on data based and behavioral based trust [10]	False Positive Rate (FPR), Detection Accuracy (DA), False Negative Rate (FNR), localization error, energy consumption	Malicious node detection consumes high computational cost. Due to indirect trust evaluation, nodes act maliciously	×	√	×
Existing models do not allow content access, reliable authentication and trust management	Blockchain authentication and trust module attains authentication and trust via digital signature [18]	N/A	Weak hashing algorithm. Poor authentication, malicious nodes tamper with the data	√	×	×
No traceability mechanism of nodes’ data fairness	BTM for malicious node detection is proposed which ensures traceability and transparency [19]	Security, traceability and reliability analysis	PoW requires high energy and faster computer processing to solve cryptographic puzzles that make it costly	×	√	×
SNs captured by malicious nodes broadcast inaccurate localization	Range free algorithm is proposed for secure localization [20]	Average localization error, localization error variance	Large communications overhead, consumes more energy due to the dynamic behavior of SNs	×	√	×
Security threats arise in IoT platform	IoT authentication protocol based on the blockchain is proposed [24]	N/A	Sink nodes do not authenticate the SNs at the time of assigning sequence numbers	√	×	√
Dynamic WSN has more uncertainty and a large coverage area, which causes trust issues	Registration of nodes, cluster formation and node logout [25]	Forward and backward security, resistance to impersonation, storage overhead, energy consumption	Complexity increases in key management. Communication overhead between BS and high storage space sensors	√	×	√
Lack of traceability of each node in the IoT network	IoT framework is proposed where tractability of each node requires nodes’ registration into the blockchain [26]	Probability of attack success, authentication accuracy	Requires extra maintenance cost and storage capacity. Data tampering in local database	×	√	√
Secure socket layer does not ensure user anonymity	The proposed system ensures data authenticity using blockchain to store data [27]	Power consumption, temperature, humidity measurement	N/A	√	×	×
Network latency and data delivery issues occur due to mobile sensors	An intrusion prevention framework is proposed for mobile IoT devices to provide reliable data routing [28]	Network lifetime, Packet Delivery Ratio (PDR), energy consumption, delay and routing overheads	In XOR hashing function, if an attacker knows one of the plain texts, then get another through them	×	√	√
Increase network overhead	Trust aware localized routing discovers multiple routes but selects one route with trusted SNs [29]	Security and throughput, encryption and decryption performance, time complexity	No authentication mechanism. Malicious nodes cause low packet delivery and high packet delay	√	√	×
Trust issues and single point of failure due to the central authority	BCR protocol is introduced that enables trust relationship between IoT vendors and cooperators [30]	Throughput, PDR, route acquisition latency, routing overhead	Low PDR	×	√	×
Malicious nodes cause gray and black hole attacks	A routing scheme through blockchain and reinforcement learning is used [31]	Enhance the routing efficiency and security of WSNs	Expense and burden increased on the server side due to the operational complexity	√	×	×
Storage and bandwidth issues	A light chain system for resource constrained devices is proposed [32]	Hash operations, hash quality, throughput, storage cost	N/A	×	×	√
Distributed nature requires high storage and faster transaction	Multi-level architecture for handling the IoUT data is proposed [33]	Reliability, accuracy, total remaining energy, energy consumption	N/A	√	×	√
Local copy of the blockchain records is not feasible	Aggregated information is used to reduce the communication cost [34]	Relative frequency, communication cost	N/A	×	×	√
Blockchain has a slow update rate, while, in Tangle, miners validate its two previous transactions before joining network	The authors presented an optimized policy by using Tangle and blockchain technologies for sampling rate [35]	Age of information and sampling interval	N/A	×	×	√
PoW requires high processing ability and data storage availability	Mobile edge computing framework is proposed to utilize the blockchain [37]	Total net revenue	N/A	√	×	√
Nodes may behave selfishly, they do not forward the packet	An incentive mechanism encourages the nodes to store the data [38]	The proposed system reduced the computing power as compared to the PoW	No authentication mechanism, expensive data storage	√	×	√
Blockchain requires high resources to perform PoW on mobile devices	Rolling blockchain is proposed where smart cars are used as the nodes of the WSN. The whole database is stored on the server [39]	Probability of finding the connected paths	Merkle tree is not utilized for this network	√	×	√
High latency, scalability issues and single point of failure	Blockchain and SDN based hybrid architecture are used [40]	Hash rate, transactions per second, average time per block and latency	Credential information stored on SDN can be leaked	×	×	√
High computational cost and storage constraint due to a large number of IoT devices	SDN, edge, fog and blockchain are used to develop a secure attack detection system [41]	F1-score, detection time, detection rate, accuracy, bandwidth Matthews correlation coefficient	System complexity increased, requires high computational power, cloud causes high latency	×	×	√
The service provider offers malicious services to the client	A blockchain based fair nonrepudiation service provisioning mechanism is proposed [42]	Average gas consumption, average transaction latency, average throughput	No off-chain mechanism is mentioned to deliver the major service part	×	√	√
No authentication, presence of malicious nodes, low PDR, high delay, usage of symmetric keys	A blockchain based authentication and trust evaluation mechanism is proposed for secure routing. RSA encryption scheme is used [Proposed Model]	Network lifetime, energy consumption, throughput, gas consumption, transaction latency, processing time of RSA encryption and processing time of trust evaluation	High time consumption in generating the RSA keys	√	√	√

Note: C1, C2 and C3 denote authentication, trust evaluation and security, respectively.

### 2.6. Data Security and Privacy

The authors in [40] propose a blockchain based hybrid network using a blockchain and software defined network (SDN) where a smart city is categorized into two groups. One is locally centralized, which is called the edge network, while the other is globally distributed, which is called the core of the network. The edge nodes preprocess the raw data. The filtered data are transmitted to the network. Devices in the network make decisions, verify the transactions and perform mining. In [41], the authors propose a secure decentralized architecture, which comprises fog, edge and SDN. A blockchain is also used to develop a secure attack detection system. Furthermore, this proposed system is implemented on the Ethereum blockchain to detect various attacks at the fog layer and maximize the attack detection on the edge layer using a deep learning algorithm. Moreover, the authors in [43] propose a tool, named AVR-INJECT, to automatically inject faults in the WSNs. This tool is time efficient and helps in analyzing the reaction and mechanism of different networks to deal with these faults.

### 2.7. Nonrepudiation Mechanism

Authors have proposed a blockchain based nonrepudiation mechanism for service provisioning. In this scheme, the blockchain acts as an evidence recorder of clients and service providers. Service programs are divided into two nonexecutable segments and delivered via on-chain and off-chain, which lessen the burden on the blockchain and avoid program disclosure [42].

## 3. Problem Statement

Recently, WSNs have contributed immensely in the development of many domains such as industrial surveillance, the military and healthcare. However, the networks encounter different challenging issues. Therefore, the authors in [19] used a blockchain based model to detect malicious nodes. However, the model has a high computational cost because the PoW consensus mechanism is used. Moreover, the nodes’ authentication is not performed, which allows unauthorized nodes to access and utilize the network resources. A blockchain based trust model is proposed in [10] on the basis of data and behavioral based trust. However, in data based trust, indirect trust is evaluated through the recommender nodes. When the recommender nodes are malicious, wrong information is provided about the legitimate nodes in the network. A routing algorithm for the WSN to find the secure route is proposed. However, the authors do not consider the malicious nodes’ detection and authentication [29]. The malicious nodes forge the actual identities of the benign nodes and drop the packets that result in a low Packet Delivery Ratio (PDR). Additionally, lifetime of the network is badly affected due to the high consumption of energy in forwarding the data packets to neighbors. Moreover, a symmetric key is used for packet encryption and decryption. However, using the symmetric key, a third party can easily gain access to the encryption key and use it for the decryption of the packets to obtain the original data. An authentication protocol for the IoT network is proposed to authenticate the nodes. The BS assigns a sequence number to every SN when it receives acknowledgment of the message. However, the BS does not verify the credentials of SNs during the assignment of sequence numbers [24]. As a result, this increases the chances of the malicious nodes to become part of the network.

## 4. System Model

We propose a secure routing mechanism in WSNs using a blockchain based encryption and trust evaluation model, motivated by [19,44]. Some assumptions of this paper are as follows.

- All the ANs, SNs and BSs have a particular Ethereum address.
- All the BSs and ANs are legitimate.
- There are no external factors and harsh network conditions that can affect the objective parameters: DT, FR and RT.

**Figure 1:**
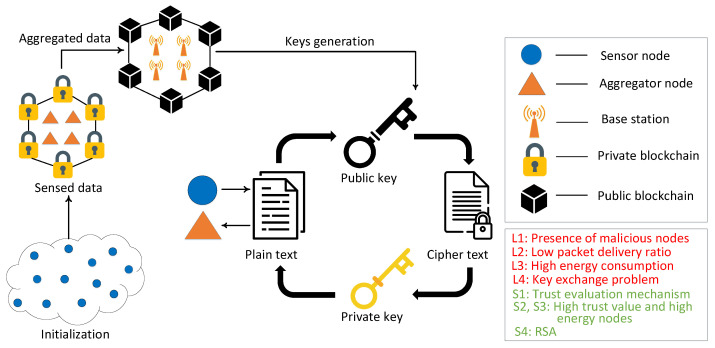
Proposed system model.

This proposed model is an extension of our work in [45]. In the proposed blockchain based routing and trust evaluation mechanism, the encrypted information of routing and trust values is transferred from BSs to other nodes in the network. Moreover, all the transactions between nodes are validated through consensus mechanisms of blockchain. Whenever the SNs communicate with the ANs, the ANs authenticate and authorize the SNs and allow them to send packets to ANs. Furthermore, when ANs want to communicate with other ANs or BSs, the BSs authenticate the ANs. After the validation of nodes’ identities, the transactions are added to the blockchain. The transaction data cannot be deleted from blockchain. When any malicious node manipulates the routing data or trust value, it is easily detected by utilizing the properties of the Merkle tree structure. The proposed model identifies the malicious nodes due to the traceability and transparency features of the blockchain. In this way, the blockchain provides secure routing and an efficient trust evaluation mechanism for malicious node detection. The proposed work uses an RSA technique to secure and reliably transmit data in the network, while in the work done in [45], enhancing the security of the transmission data is not considered. In the proposed model, initially, the data are sensed by the SNs and sent to the associated ANs. Afterwards, the ANs receive and forward the data to the nearest BSs. Two blockchains are used in our model to register and authenticate different nodes in the network. Ethereum is used for implementing the PoW and PoA consensus mechanisms by ropsten and rinkeby test networks. However, in the previous model of Bitcoin, a smart contract was not introduced. Ethereum introduces the smart contract that is a self-executing agreement that executes when predefined operations are met. A smart contract helps in eliminating the need for a third party and the associated risks. This is the reason that Ethereum is used for the blockchain in our proposed model. The public blockchain is deployed on the BSs that register and authenticate the ANs. Moreover, the BSs authenticate the communication between the ANs. The identities of the ANs are stored on the public blockchain, and they are allowed to join and access the private blockchain. Furthermore, ANs that are a part of the private blockchain perform the registration and authentication of the SNs, as shown in Figure 1.

Private and public blockchains are used for authentication of SNs and ANs. Each SN can only be a part of one cluster network. Afterwards, the SNs broadcast the request message of registration ($SN_{ID}$, $AN_{ID}$ and $BS_{ID}$). The smart contract deployed for the SNs’ registration process is triggered by the registration event in the private blockchain. Two types of blockchains are used in this model to minimize the workload of ANs. In the previous authentication scheme, the ANs are responsible for registering and authenticating other ANs [44], due to which they die in initial rounds. However, in our proposed model, ANs are registered and authenticated by BSs, which have high computational resources. In this way, the workload of ANs is reduced. Hence, both blockchains coexist to reduce the computational overhead of the proposed model. The identification of all nodes is uploaded on the public blockchain as the ANs are directly connected with the public blockchain. Furthermore, different features of the blockchain that make our model efficient are given below.

- PoA consensus algorithm is used in the private blockchain for validation of transactions and adding the blocks into the blockchain.
- PoW consensus algorithm is used in the public blockchain to validate the transactions and add the blocks into the blockchain.
- Mutual authentication: When two nodes want to communicate with each other, they first need to be recognized before the interaction. The identity of all nodes is stored on the BSs that authenticate the ANs.
- Nonrepudiation: The nodes that take part in the communication cannot deny sending the packets. The nonrepudiation scheme is performed on the blockchain. All operations are stored on it, therefore, data tampering cannot be performed.
- Integrity: This includes the data packets’ integrity, where unauthorized nodes cannot access and illegally tamper with the data packets in the interaction process. The integrity of the data packets is ensured by the authentication process, which is carried out by the public and private blockchains.
- Transparency and traceability: The whole process is traceable and transparent because the information of SNs is bound to each data record. Whenever any malicious node exists in the WSN, it can be identified by the traceability feature of the proposed model.

The steps included in the proposed model are initialization, registration, authentication and trust evaluation of the nodes.

### 4.1. Initialization

In this step, all existing nodes of the network are initialized. In the proposed model, RSA is used for securing data transmission. It is composed of three processes: Key generation, encryption and decryption. Each node generates the public and private keys for itself. The public key of every node is stored on the BS, where public blockchain is deployed, while the private key of each node is kept secret and is only known by the authorized node. Moreover, it is assumed in the proposed work that both BSs and ANs are the trusted nodes and ANs aggregate and forward the data. The SNs encrypt the collected information with the public keys of ANs and send it to the ANs. The ANs decrypt the data packets with their private keys. The same process is performed when sending the data packets from ANs to BSs.

### 4.2. Registration

All nodes have unique Media Access Control (MAC) addresses. The identity of the SNs, ANs and BSs is marked as $SN_{ID}$, $AN_{ID}$ and $BS_{ID}$, respectively. The ANs are registered using the smart contract of the public blockchain. The smart contract verifies the existence of ANs. Moreover, the validity of ANs’ identities and MAC addresses is also checked by the BSs. In the registration process, the public blockchain keeps a record of ANs’ identities when the above steps are successfully performed. When the identities of ANs are stored in the blockchain, no one can maliciously tamper with these identities. In this way, the blockchain provides a reliable authentication mechanism in our WSN. In contrast, when the verification of ANs’ identities fails, then these ANs are revoked from the network. After completing the registration process, SNs are permitted to join the blockchain. The SNs, after their deployment, are associated with the corresponding ANs. External attacks are reduced by registering the nodes.

### 4.3. Authentication

When SNs communicate with ANs, the ANs authenticate the identities of SNs by utilizing a private blockchain. Furthermore, when ANs communicate with BSs, the BSs authenticate them by exploiting a public blockchain. When two ANs communicate with each other, they send requests to the BSs, therefore, mutual authentication between ANs is performed.

The procedures of nodes’ registration, mutual authentication and trust evaluation are described in Algorithms 1–3, respectively.
**Algorithm 1:**
Nodes’ registration

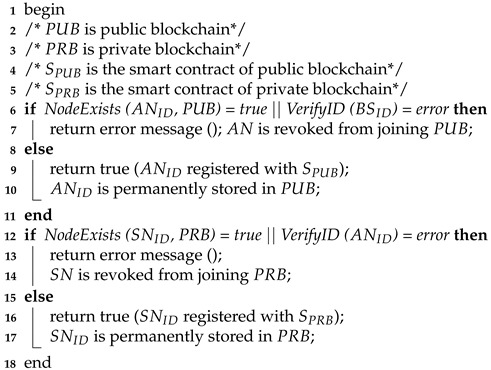


### 4.4. Trust Evaluation Mechanism

The traditional systems lead to issues such as lack of trust, single point of failure, high computational cost, etc. The SNs may behave selfishly in the networks due to the limited resources. Furthermore, any malicious node can become part of the network and perform malicious activities. To tackle these issues, a blockchain is used with our trust evaluation mechanism. The trust value of all nodes is calculated by considering the Forwarding Rate (FR), Response Time (RT) and Delayed Transmission (DT). After the trust evaluation, the trust values are stored in the blockchain. The blockchain provides data immutability wherein data cannot be tampered with by the malicious nodes. Moreover, no node can repudiate its action as the blockchain provides traceability in the trust evaluation process. Therefore, the trust value of each SN is calculated to remove selfish and malicious nodes from the network. The trust value of each SN is compared with a predefined threshold and then malicious and legitimate nodes are classified considering this threshold, motivated by [19]. We have considered legitimate and malicious nodes in the network and assumed that there are no external factors and harsh environmental conditions that can affect the objective parameters: FR, RT and DT. This means that the node cannot be a malfunctioning node due to its internal technical faults and harsh network conditions. Moreover, the trust value is calculated considering DT, FR and RT, which are completely dependent on the behavior of SNs. This research assumption is supported by the works of [46,47,48] The steps necessary for the trust evaluation of the SNs are mentioned below.

Step 1: ANs determine the states of SNs as either alive or dead.

Step 2: The Node Communication Quality (NCQ) is computed for the alive SNs on the basis of DT, FR and RT.

Step 3: The number of successful and unsuccessful communications is computed based on the NCQ. When the value of the NCQ is greater than the threshold, it is considered as a successful communication, otherwise as a unsuccessful communication.

Step 4: The trust values of the SNs are determined on the basis of successful and unsuccessful communications.

Step 5: When the trust values of SNs are greater than the threshold, they are considered as legitimate nodes, otherwise malicious nodes.

Step 6: After the trust evaluation, the ANs send the trust values of the SNs to the BSs and malicious nodes are removed from the network.
**Algorithm 2:**
Mutual authentication

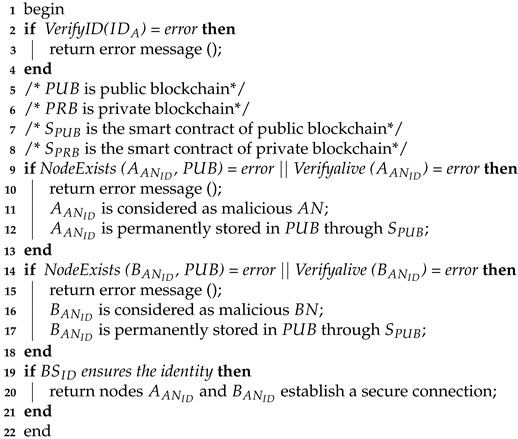


In our model, the routing is improved by the trust evaluation mechanism in our system model. First of all, the trust value of each node is calculated considering DT, RT and FR. After the calculation of the trust value, the nodes with a trust value lower than the predefined threshold are considered as malicious nodes and removed from the network. So, only legitimate nodes remain in the network and participate in the routing mechanism. In this way, the routing mechanism is improved by a trust evaluation mechanism.

In the network, the SNs are divided into alive and dead nodes. When the SNs are dead, they are removed from the network, otherwise the following factors are used in the trust evaluation of the SNs.

#### 4.4.1. Delayed Transmission

This is the time required to send the data packets from the source to the destination. It is computed by the following equation [19]:

(1) $$\begin{array}{c} DT=\frac{Tsensor_{id}}{T_{1}}*100\%,\;0<\frac{Tsensor_{id}}{T_{1}}<1, \end{array}$$

where $Tsensor_{id}$ and $T_{1}$ denote the time required to forward the data packets after receiving them and the time interval in which the data packet is sent from the source to the destination, respectively.
**Algorithm 3:**
Trust value evaluation of sensor nodes
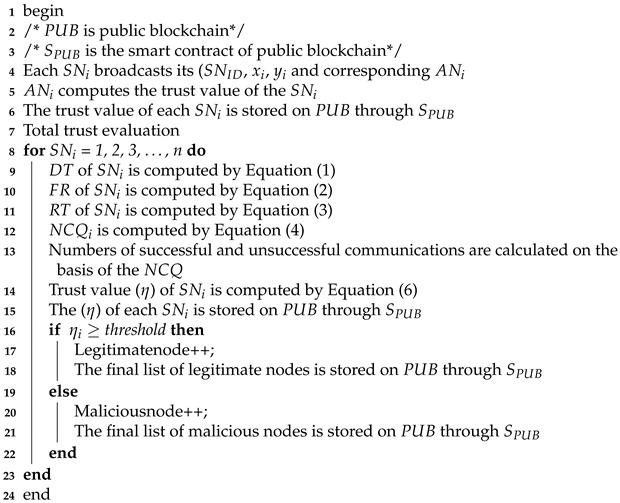


#### 4.4.2. Forwarding Rate

This is used to evaluate the integrity of the data packets to avoid data tampering by the malicious nodes. It is calculated as the ratio of packets received by the ANs to the data packets sent by the SNs [19].

(2) $$\begin{array}{c} FR=\frac{td}{sd}, \end{array}$$

where *td* and *sd* are the packets received by the ANs and packets sent by the SNs, respectively.

#### 4.4.3. Response Time

This refers to the total time between request initialization and its fulfillment. It is computed by the following equation [19]:

(3) $$\begin{array}{c} RT=\frac{\frac{dbn}{bw}+\frac{pd}{ps}+pt}{T_{2}}, \end{array}$$

where *dbn*, *pd*, *pt*, *bw*, *ps* and $T_{2}$ are the number of packet bits, propagation distance, processing time, network bandwidth, propagation speed and time interval, respectively.

#### 4.4.4. Node Communication Quality

NCQ helps in calculating the number of successful and unsuccessful communications on the basis of DT, FR and RT. It is computed by the following equation [19]:

(4) $$\begin{array}{c} NCQ=\gamma *DT+\lambda *(1-FR)+\sigma *RT, \end{array}$$

where the weights for *DT*, *FR* and *RT* are γ, λ and σ, respectively [19].

(5) $$\begin{array}{c} \gamma +\lambda +\sigma =1. \end{array}$$

The weights are adjusted for the proposed model that shows the importance of *DT*, *FR* and *RT*. The weights considered for the trust evaluation are γ = 0.33, λ = 0.34 and σ = 0.33, which are taken from [19].

The threshold κ is set as per the following scenario. When NCQ >κ, the number of unsuccessful communications $N_{F}$ of the SNs is increased. Otherwise, the number of successful communications $N_{S}$ of the SNs is increased. The trust values of the SNs are computed using the successful and unsuccessful communications by the following equation [19]:

(6) $$\begin{array}{cc} \eta & =\frac{N_{S}+1}{N_{S}+N_{F}+2}. \end{array}$$

After the calculation of the trust values, the trust value of each node is compared with a predefined threshold. The nodes with a trust value higher than threshold are considered as legitimate nodes, otherwise they are considered as malicious nodes. In Table 2, the mapping between identified limitations, proposed solutions and validations is shown. The first limitation (L1) is the presence of malicious nodes, which degrades the network’s performance. To solve this issue, the trust value of each node is computed that depends on DT, FR and RT and it is used to differentiate between the malicious and legitimate nodes. The FPR, FNR and DA are used to evaluate the proposed model. The second and third limitations (L2 and L3) are low PDR and high energy consumption. After the trust evaluation, the SNs with high trust values take part in the packet transmission, which increases the PDR. The ANs collect data from the corresponding SNs and forward them to the BSs. The performance parameters, i.e., PDR, network lifetime and residual energy, are used to check the validity of the proposed model. The fourth limitation (L4) is a key exchange problem. To deal with this limitation, RSA is used for packet encryption and decryption.

## 5. Simulation Results

In this section, the evaluation of the proposed model through simulation is discussed. In our proposed model, the malicious SNs in the network are identified through a trust evaluation mechanism. Moreover, an authentication scheme is provided to secure our network from intrusion. Furthermore, a routing mechanism is proposed that ensures real time energy efficient data delivery from SNs to BSs. The proposed model is compared with the existing model on the basis of authentication. As the effect of authentication cannot be visualized directly, it is evaluated on the basis of network lifetime, energy consumption and throughput. Moreover, Solidity is used to write the smart contract. The overall network is validated using both PoW and PoA consensus algorithms. Gas consumption and transaction latency are the parameters through which the implemented algorithms are compared [42].

### 5.1. Simulation Setup

The specifications for the simulation setup include an Intel(R) Core (TM) i5-5200U CPU @ 2.20 GHz, 8 GB RAM and a 64-bit operating system, (Santa Clara, California, United States). For performing the simulations, SNs are stationary. The simulation parameters of our model evaluation are given in Table 3.

### 5.2. Performance Metrics

Different performance metrics are considered for the proposed system evaluation, which are given below.

#### 5.2.1. Packet Delivery Ratio

This represents the ratio of data packets successfully received at the BSs to the data packets sent by the SNs.

#### 5.2.2. Network Lifetime

This is the time period in which the network is operational. The network lifetime depends on the number of alive nodes.

#### 5.2.3. Residual Energy

To analyze the energy consumption of SNs, the residual energy is considered in each round. As the number of rounds is increased, the residual energy of the SNs is decreased. If the energy of an SN is less than a specific threshold, then this SN is broadcast as a dead node in the network.

#### 5.2.4. False Positive Rate

This is defined as the number of all honest nodes that are identified as malicious nodes by the proposed model. It is computed by the following equation [10]:

(7) $$\begin{array}{c} FPR=\frac{FP}{FP+TN}, \end{array}$$

where *FP* and *TN* are the false positives and true negatives, respectively.

#### 5.2.5. False Negative Rate

The is defined as the number of all malicious nodes that are identified as legitimate nodes by the proposed model. It is computed by the following equation [10]:

(8) $$\begin{array}{c} FNR=\frac{FN}{FN+TP}, \end{array}$$

where *FN* and *TP* are the false negatives and true positives, respectively.

#### 5.2.6. Detection Accuracy

This is defined as the ratio of trusted SNs identified as malicious nodes to the total number of malicious nodes in the entire network. It is computed by the following equation [10]:

(9) $$\begin{array}{c} DA=\frac{M_{identified}}{M_{total}}. \end{array}$$

**Figure 2:**
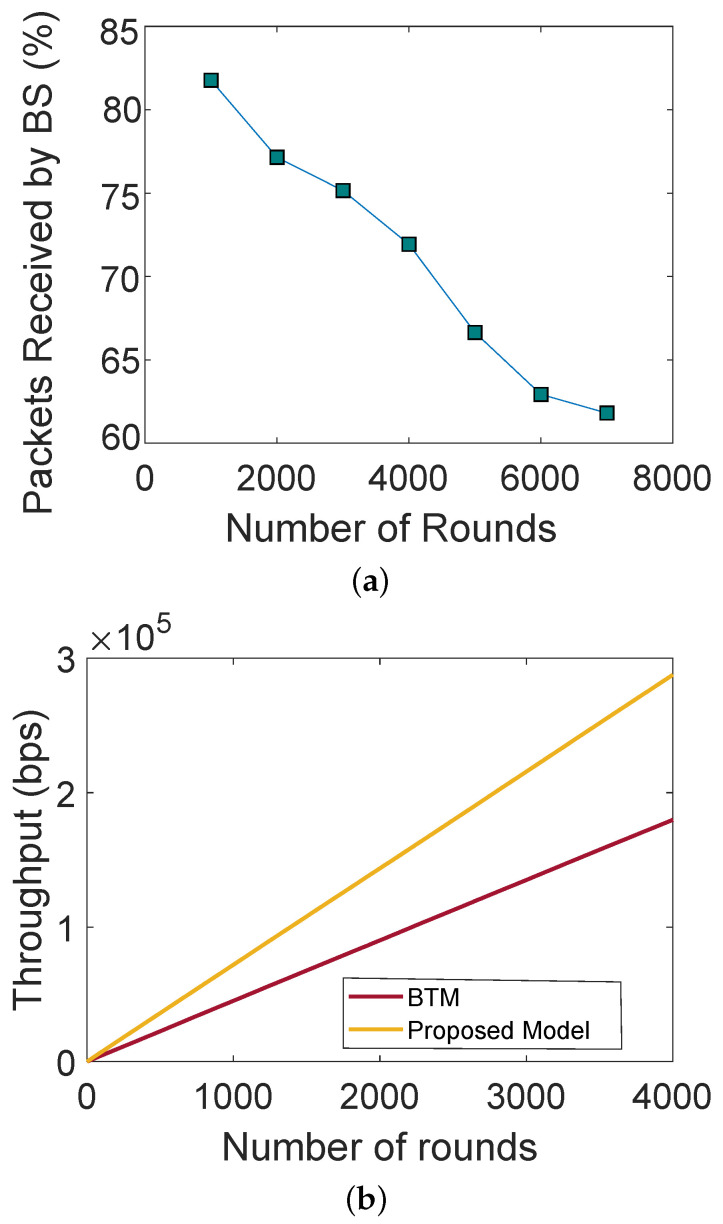
(**a**) Packet delivery ratio, (**b**) throughput.

Figure 2a shows the PDR with respect to the number of rounds. The number of data packets decreases with an increasing number of rounds. A large number of SNs participate in packet transmission, which reduces the computational overhead of a single SN. The proposed model increases the probability of the data packets being received successfully. Therefore, there is a high value of PDR in an initial round. As long as all SNs remain alive, they send more packets to the BSs. Moreover, the figure depicts the PDR with respect to round number. The SNs are not enriched with energy because they have limited batteries. Therefore, they are more likely to die as the number of rounds increases, due to which PDR decreases. Furthermore, Figure 2b shows the network throughput with respect to authenticated and nonauthenticated nodes. In a network where no authentication is performed, any malicious node can become a part of the network and perform malicious activities, whereas, in a network with authentication, only registered and authentic nodes become part of the network. The figure shows that the throughput of the network gradually increases as the number of rounds increases.

**Figure 3:**
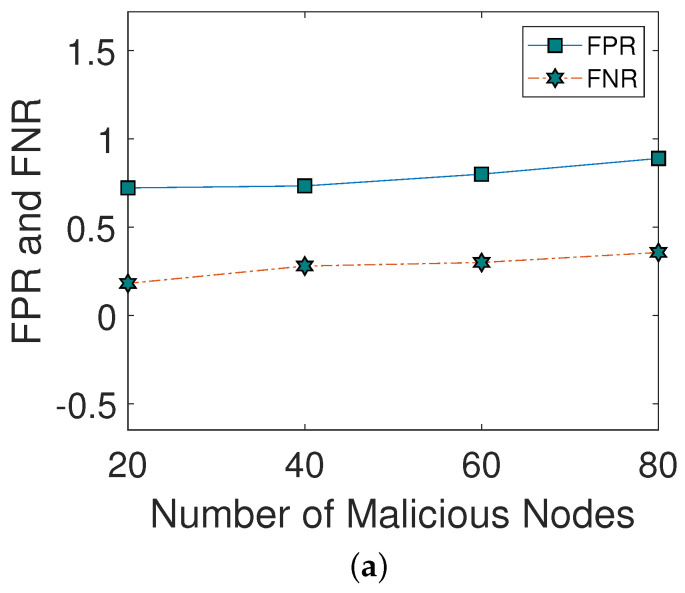

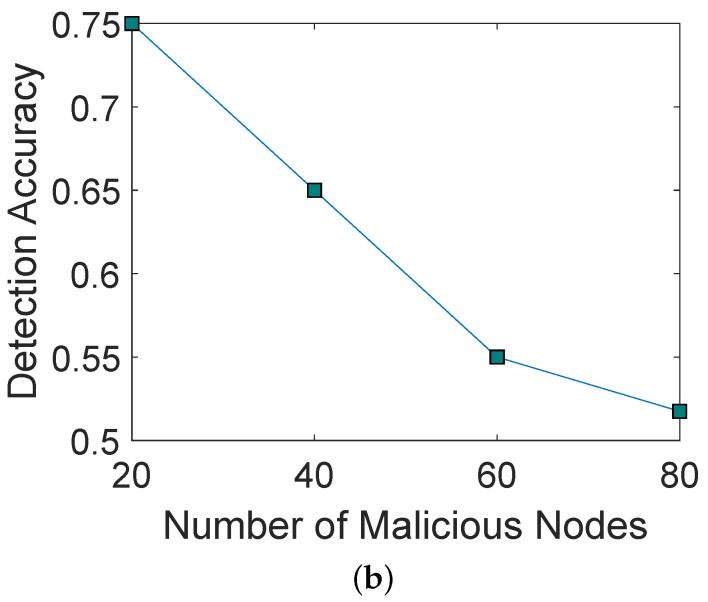
(**a**) FPR and FNR, (**b**) detection accuracy.

Figure 3a shows the impact of different malicious nodes on FPR and FNR. As the number of malicious nodes increases, FPR and FNR also increase. The reason is that a large number of malicious nodes broadcast a large amount of wrong information in the network. Figure 3b illustrates the impact of different malicious nodes on the DA. As the number of malicious nodes is increased, the DA of the network is decreased. Moreover, when the number of malicious nodes is greater than 20, DA is decreased due to greater FPR and FNR.

**Figure 4:**
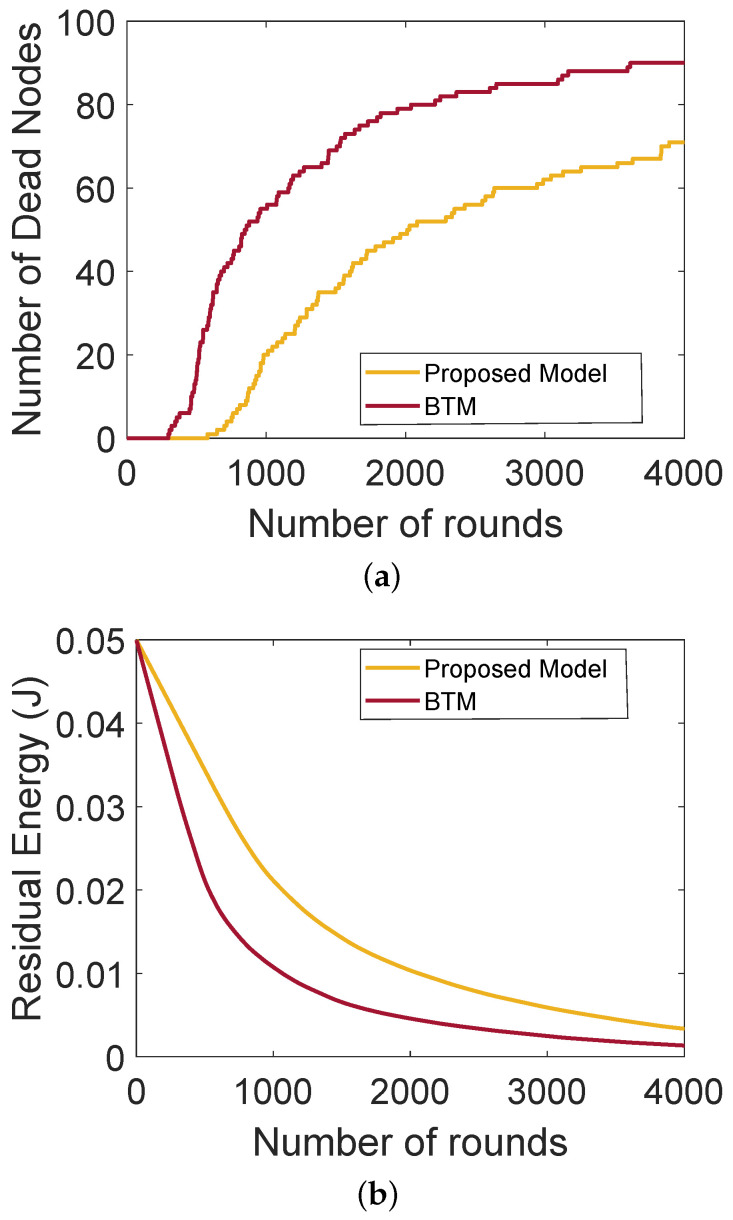
(**a**) Number of dead nodes with rounds, (**b**) residual energy of the nodes.

Figure 4a shows the comparison of the network lifetime of the proposed model with BTM. It also depicts the network stability period and shows that SNs do not communicate directly with the BSs due to the long distance between them. In the network BTM, no authentication mechanism is performed, thus malicious nodes can join the network, impersonate the identity of the legitimate nodes and transmit wrong information, which affects the network performance. In the proposed model, authentication of nodes is performed. Therefore, external malicious nodes are not allowed to become part of the network. After the authentication, the nodes behaving selfishly are detected on the basis of their trust values and then removed from the network. Thus, the network lifetime of the proposed model outperforms that of BTM. Figure 4b shows the lifetime of a network that depends on the residual energy of the SNs. These figures only depict the operability of our proposed model and the blockchain mechanism has no impact on the stability period and residual energy. The blockchain provides transparency in the network about the interactions taking place between nodes, which further helps in avoiding the interception of the external nodes. Moreover, only those nodes take part in the network communication, which are being authenticated in the first place. In the BTM with no authentication, the malicious nodes participate in the network and send excessive amount of wrong information to the forwarder nodes that consume a lot of energy while transferring the packets, while little energy is consumed in the proposed model.

**Figure 5:**
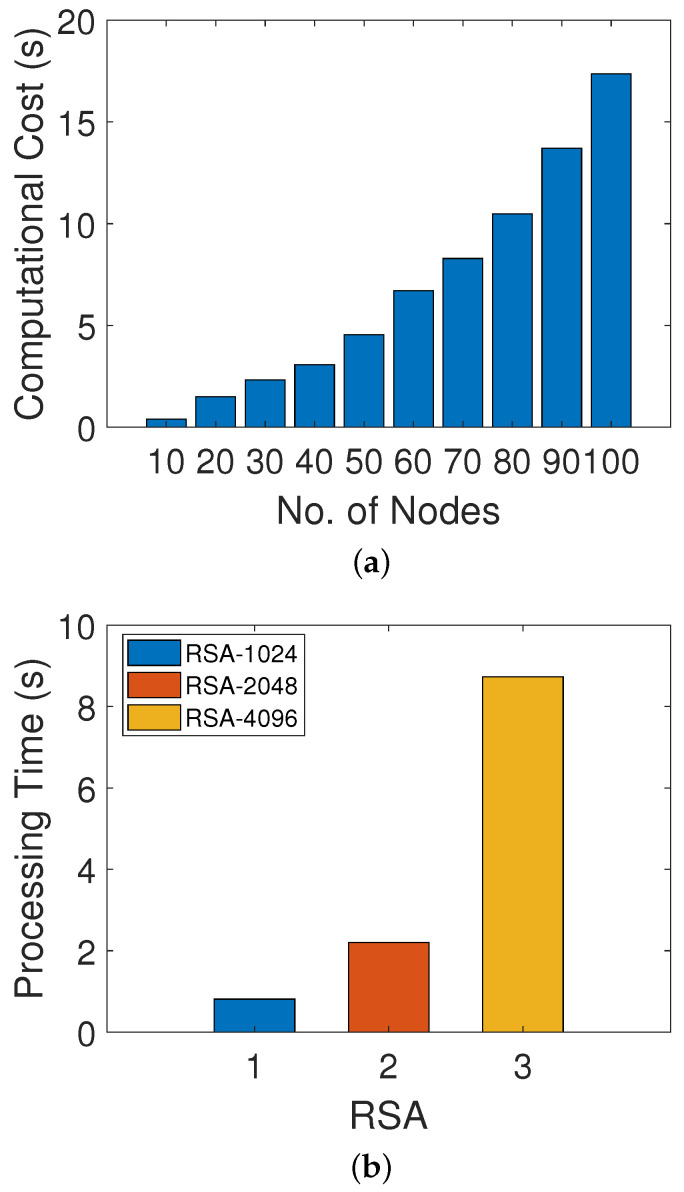
(**a**) Processing time of trust evaluation, (**b**) processing time of RSA encryption.

Figure 5a shows the amount of time consumed in trust evaluation of different numbers of nodes. It is clear from the figure that the time taken in calculating the computational cost in the trust evaluation of 10 nodes is 0.39 ms while, for 100 nodes, the computational cost is 17.37 ms. The trust evaluation mechanism is performed by considering DT, FR and RT values, which are completely dependent upon the behavior of a node. Moreover, it is clear from the figure that the computational cost of the network increases as the trust evaluation increases with the increasing number of nodes. Figure 5b shows the amount of time that the RSA technique takes in the generation of public and private keys with respect to different key lengths. The time taken to generate a key of 1024 bits takes less time, that is, 0.81 s, while the generation of keys with 2048 bits and 4097 bits takes 2.20 s and 8.73 s, respectively. RSA with a large bit key size takes a lot of time in key generation and provides more security than other schemes. Moreover, there is a tradeoff between security and performance time, as, while achieving high security, time will be compromised.

**Figure 6:**
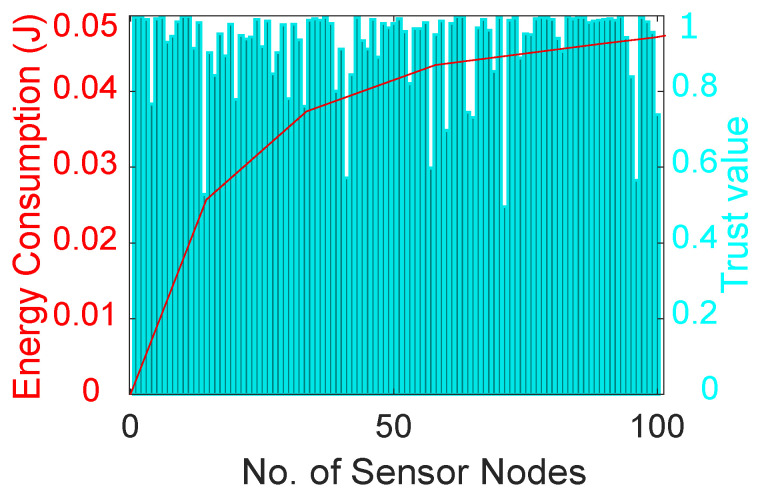
Energy consumption in trust evaluation of nodes.

Figure 6 shows the trust values of different nodes that are calculated considering three performance metrics: DT, FR and RT. Different nodes have different trust values due to their performance in the network. The trust value of each node is compared with a predefined threshold and nodes with high trust values are considered legitimate nodes, otherwise they are considered malicious nodes. Moreover, the figure depicts the amount of energy consumed in the calculation of the trust value of different nodes in the network. The amounts of energy consumed for calculating DT, FR and RT are 0.00165 J, 0.0017 J and 0.00165 J, respectively. Furthermore, the amount of total energy consumed in the network increases when the number of nodes increases. After some time, the overall energy consumption of the network shows a gradual increase until it becomes almost constant.

**Figure 7:**
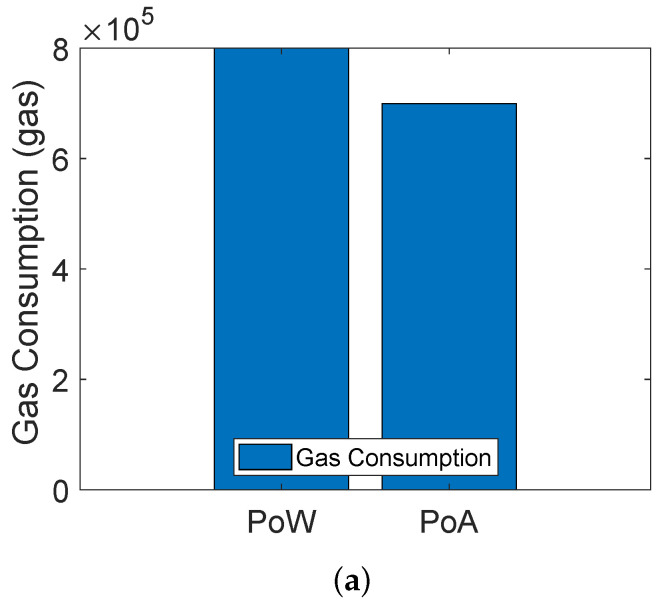

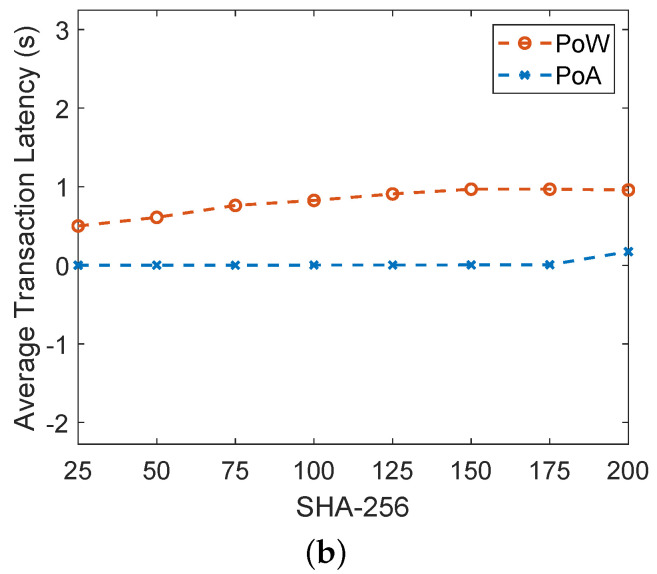
(**a**) Comparison of gas consumption between PoA and PoW, (**b**) comparison of average transaction latency between PoA and PoW.

The blockchain based trust evaluation model of [19] uses the PoW consensus mechanism for validating transactions and adding blocks into the blockchain. On the other hand, we use both PoA and PoW in our proposed model. Figure 7a shows the comparison between PoA and PoW consensus mechanisms. The comparison is shown in terms of gas consumption. When any transaction is performed in Solidity, a fixed amount of gas is consumed against it. The unit of gas consumption in Solidity is Gwei and 1 Gwei≈0.000000001 ether. Moreover, the cost of 1 Ethereum is approximately equal to USD 3825.16. It is shown in the figure that gas consumption of PoW is more compared to PoA. The reason is that PoW becomes computationally expensive due to the participation of all miner nodes in solving the complex mathematical puzzle. The node that solves the puzzle first is responsible for validating the transaction and adding the block into the blockchain. On the other hand, no puzzle solving is involved in PoA, because there are preselected validators that validate the transaction and add blocks into the blockchain. Although the PoW is costly in terms of monetary cost as compared to PoA, the PoW consensus mechanism is implemented on the public blockchain, which is connected with many other private blockchains and it is more likely to be attacked by the malicious nodes. Therefore, we have considered the PoW. In the case of a private blockchain, we use the PoA because the environment is private and only authenticated nodes exist. So, ultimately, there is a tradeoff between the cost and security. Moreover, Figure 7b shows the average transaction latency of both the PoW and PoA consensus mechanisms. PoA has low transaction latency as compared to PoW. In PoA, the miners are preselected nodes that do not perform mathematical puzzles, which consumes high computational power, as in the case of PoW.

## 6. Formal Security Analysis

In this section, the security analysis of the smart contract is performed through the Oyente [49], whereas the sybil and Denial of Service (DoS) attack detection mechanism is performed in the main network. The smart contract is vulnerable to different attacks such as integer overflow, underflow, parity multisig bug 2, transaction ordering dependence, timestamp dependency, callstack depth attack and re-entrancy [50]. These attacks are defined as follows.

### 6.1. Integer Underflow and Overflow

The integer underflow occurs when a variable is decremented until it is below the minimum value, while integer overflow occurs when it exceeds the maximum value. In both cases, the operability of the network is affected. The minimum and maximum values of the unsigned integers lie between 0 and 32 bytes.

### 6.2. Parity Multisig Bug 2

Parity multisig is used by the account’s users to manage the digital assets, which contain the data of withdrawal voting, the daily limit of withdrawal and ownership information stored in the users’ accounts. The information is publicly accessible to other entities in the network. However, the attacker accesses the account of a victim due to the centralized system and generates fake signatures.

### 6.3. Callstack Depth Attack Vulnerability

The smart contract invokes other smart contracts via some external functions such as call (), transfer (), etc. In the external virtual machine, the limit of the smart contract frame is 1024. When the limit exceeds 1024 frames, the external virtual machine triggers the error.

### 6.4. Transaction Ordering Dependence

This is the process of carrying out transactions that are based on the required amount of gas. The price of gas determines which transaction must be mined first. However, the attacker modifies the gas price during its transaction.

### 6.5. Re-Entrancy Vulnerability

This error occurs when repeated calls to the same function are made numerous times, due to which the function cannot be executed. The transactions wait for the current call to finish before responding to the next one.

### 6.6. Timestamp Dependency

**Figure 8:**
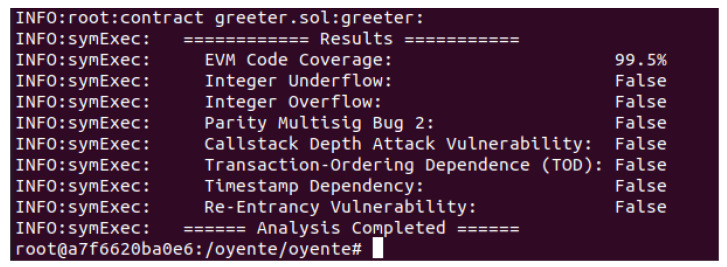
Formal analysis of smart contract using Oyente.

The attackers manipulate timestamps to gain control over the mining process. Every transaction has a timestamp, which is vulnerable to tampering. The smart contract used in the proposed model is analyzed against the aforementioned vulnerabilities, as shown in Figure 8. The results for all vulnerabilities are false, which show that the smart contract is secure and robust against all vulnerabilities.

## 7. Attacker Model

A variety of attacks are possible in the WSN, such as sybil and DoS attacks. In a sybil attack, the malicious nodes steal the identity of legitimate nodes or illegally create fake multiple identities and then become part of the network. The malicious nodes eavesdrop on the communication between nodes and then broadcast wrong information into the network. The attack is performed in multiple ways. One of them is direct communication between the legitimate and malicious nodes. Another way is when a malicious node steals the identity of a legitimate node and broadcasts wrong information on the behalf of legitimate nodes [49]. Finally, another attack is the DoS attack, where the malicious nodes launch an attack to exhaust the energy of legitimate nodes. This attack affects the routing and performance of the network.

**Figure 9:**
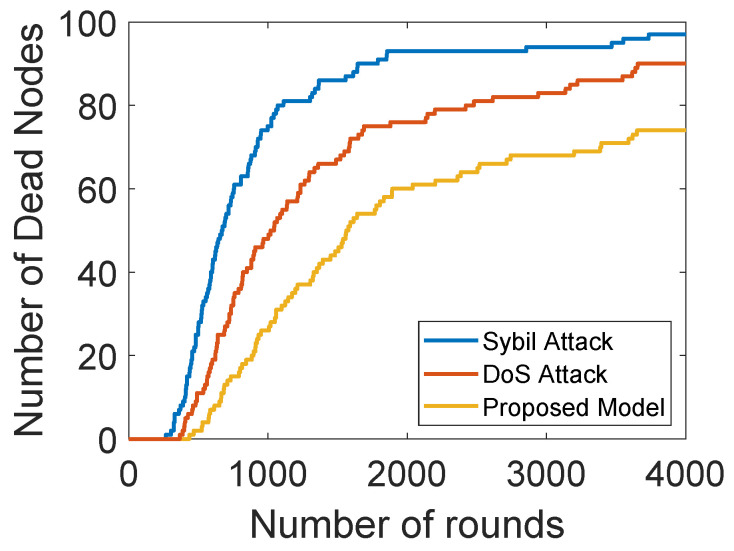
Dead nodes with and without attacks.

Figure 9 shows the comparison of network lifetimes of our proposed model with and without sybil and DoS attacks. The network lifetime of the proposed solution is longer than the model with DoS and sybil attacks. In a sybil attack, the malicious nodes create multiple fake identities or steal the IDs of the legitimate nodes. After this, they claim themselves as the legitimate nodes to disrupt the whole network. As the sybil attacker has multiple identities, they send multiple packets with wrong information to ANs through multiple identifiers. In a DoS attack, the malicious nodes steal the IDs of legitimate node and then forward unwanted information towards ANs. A lot of energy of ANs is consumed in discarding unwanted information, which degrades the network lifetime. Moreover, communication with the malicious nodes results in a data loss. Therefore, an authentication and trust evaluation mechanism is proposed in this model. Firstly, the nodes are authenticated through their MAC addresses. Then, the trust of each SNs is evaluated on the basis of FR, RT and DT. After this, the NCQ value of each SN is computed and nodes with a trust value lower than the thresholds are removed from the network.

**Figure 10:**
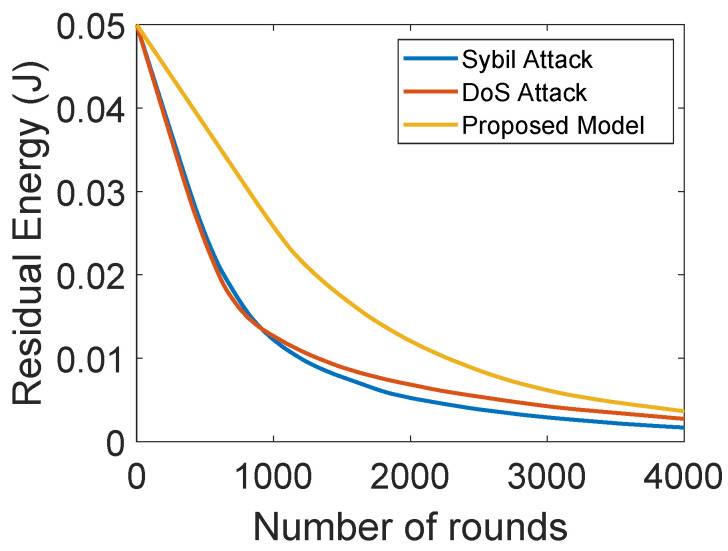
Residual energy with and without attacks.

Figure 10 shows the energy consumption of the proposed model with DoS and sybil attacks. In the sybil and DoS attacks, the attackers forge information and broadcast unwanted information that consumes a lot of energy of ANs. The proposed model outperforms the model with sybil and DoS attacks. In the proposed model, each SN forwards the sensed information towards its associated AN. The ANs compute trust values of SNs on the basis of DT, FR and RT. According to the value of NCQ, the number of successful and unsuccessful communications is computed. Then, the trust value is computed, and the SNs with trust values below the threshold are removed from the network. Therefore, minimal energy is consumed in the proposed model, which indicates the maximum network lifetime.

**Figure 11:**
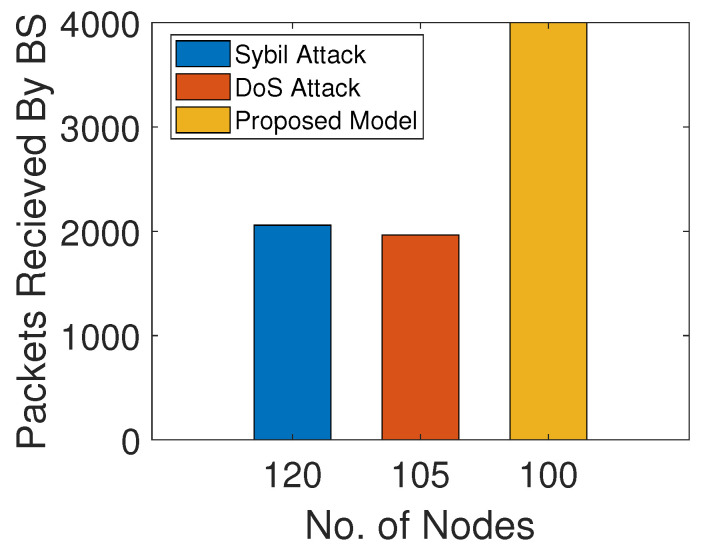
PDR with and without attacks.

Figure 11 shows PDR analysis of the model with the sybil and DoS attacks and the network without these attacks. In the presence of sybil and DoS attackers, few packets are received at the BS. The attackers selectively forward or tamper with the packets. In the proposed model, the trust values of the SNs are computed by the ANs. Then, only trusted SNs participate in forwarding the data packets. Hence, high PDR indicates minimum packet loss in the proposed model.

## 8. Conclusions and Future Work

A blockchained secure routing mechanism for WSNs is proposed in this paper. The SNs and ANs are authenticated using private and public blockchains, respectively. The private blockchain is deployed on ANs while the public blockchain is deployed on the BSs. The trust values of SNs are computed on the basis of DT, FR and RT after the SNs’ authentication. The SNs with high trust values are considered as legitimate while others are considered as malicious. The simulation results show that when there are a large number of trusted SNs in the network, the PDR of the network is high. As the energy of SNs gradually depletes, they start to die and only a few SNs participate in the network. As a result, PDR is decreased. Furthermore, FPR and FNR are increased in the presence of malicious nodes that have a negative impact on the DA. The DA is decreased due to high FPR and FNR. Furthermore, RSA is used for the encryption and decryption of the data packets for secure routing. In the future, we will implement the proposed work in different real world networks.

## Figures and Tables

**Table 2:** Mapping table of limitations, their solutions and the validation parameters.

Identified Limitations	Proposed Solutions	Validation Done
L1: Presence of malicious nodes	S1: Trust evaluation considering NCQ value to remove malicious nodes from the network	V1: Trust values of the SNs, FNR, FPR and DA. The results are depicted in Figure 2b and Figure 3a,b
L2: Low PDR due to the involvement of malicious nodes L.3: High energy consumption of the SNs	S2, S3: The trusted SNs perform routing. SNs send their packets to the ANs, who forward the packets to BSs. Through this process, little energy is consumed by the SNs	V2, V3: PDR, network lifetime and residual energy. The results are depicted in Figure 2a and Figure 4a,b
L4: Key exchange problem	S4: RSA is used for the secure transmission of data considering key generation, encryption and decryption	V4: Direct validation is not shown explicitly

**Table 3:** Simulation parameters.

Parameters	Values
Sensing area	100 × 100 m${}^{2}$
SNs	100
ANs	4
BSs	2
Deployment	Random
Initial energy of SNs	0.05 J

## Abbreviations

The following abbreviations are used in this manuscript:

ANs	Aggregator Nodes
API	Application Programming Interface
BSs	Base Stations
DA	Detection Accuracy
DT	Delayed Transmission
FR	Forwarding Rate
FNR	False Negative Rate
FPR	False Positive Rate
IoT	Internet of Things
MAC	Media Access Control
NCQ	Node Communication Quality
PDR	Packet Delivery Ratio
PoA	Proof of Authority
PoS	Proof of Stake
PoW	Proof of Work
RT	Response Time
RSA	Rivest–Shamir–Adleman
SNs	Sensor Nodes
SDN	Software Defined Network
WSNs	Wireless Sensor Networks
$AN_{ID}$	Identity of an AN
$BS_{ID}$	Identity of a BS
$SN_{ID}$	Identity of an SN
PRB	Private Blockchain
PUB	Public Blockchain
κ	Threshold to check the NCQ
η	Trust value of SNs
γ	Weight of DT
λ	Weight of FR
σ	Weight of RT
